# How Geographical Isolation and Aging in Place Can Be Accommodated Through Connected Health Stakeholder Management: Qualitative Study With Focus Groups

**DOI:** 10.2196/15976

**Published:** 2020-05-27

**Authors:** Sonia Chien-I Chen, Chenglian Liu, Zhenyuan Wang, Rodney McAdam, Michael Brennan, Shirley Davey, Teng Yuan Cheng

**Affiliations:** 1 Institute of Quantitative Economics Huaqiao University Xiamen China; 2 School of Computing Neusoft Institute of Guangdong Foshan China; 3 Faculty of Economics and Management East China Normal University Shanghai China; 4 Antai College of Economics and Management Shanghai Jiao Tong University Shanghai China; 5 Ulster Business School Ulster University Newtownabbey United Kingdom; 6 Nanjing Audit University Naijing China

**Keywords:** connected health care, remote areas, business strategy, Taiwan, population aging, knowledge sharing

## Abstract

**Background:**

In remote areas, connected health (CH) is needed, but as local resources are often scarce and the purchasing power of residents is usually poor, it is a challenge to apply CH in these settings. In this study, CH is defended as a technological solution for reshaping the direction of health care to be more proactive, preventive, and precisely targeted—and thus, more effective.

**Objective:**

The objective of this study was to explore the identity of CH stakeholders in remote areas of Taiwan and their interests and power in order to determine ideal strategies for applying CH. We aimed to explore the respective unknowns and discover insights for those facing similar issues.

**Methods:**

Qualitative research was conducted to investigate and interpret the phenomena of the aging population in a remote setting. An exploratory approach was employed involving semistructured interviews with 22 participants from 8 remote allied case studies. The interviews explored perspectives on stakeholder arrangements, including the power and interests of stakeholders and the needs of all the parties in the ecosystem.

**Results:**

Results were obtained from in-depth interviews and focus groups that included identifying the stakeholders of remote health and determining how they influence its practice, as well as how associated agreements bring competitive advantages. Stakeholders included people in government sectors, industrial players, academic researchers, end users, and their associates who described their perspectives on their power and interests in remote health service delivery. Specific facilitators of and barriers to effective delivery were identified. A number of themes, such as government interests and power of decision making, were corroborated across rural and remote services. These themes were broadly grouped into the disclosure of conflicts of interest, asymmetry in decision making, and data development for risk assessment.

**Conclusions:**

This study contributes to current knowledge by exploring the features of CH in remote areas and investigating its implementation from the perspectives of stakeholder management. It offers insights into managing remote health through a CH platform, which can be used for preliminary quantitative research. Consequently, these findings could help to more effectively facilitate diverse stakeholder engagement for health information sharing and social interaction.

## Introduction

### Background

The global phenomenon of an aging populations has made health care a universal issue, especially in remote areas. Connected health (CH) has been proposed as a promising solution to manage challenges arising in these remote areas. It aims to reduce isolation, enhance safety, increase efficiency, and diminish costs for remote residents [1]. It acts as an alternative solution to make “aging in place” feasible by connecting infrastructure, devices, and health care stakeholders [2]. In remote areas, CH is needed, but as local resources are often scarce and the purchasing power of residents is usually poor, it is a challenge to apply CH in this setting. In this study, remote areas were defined in 3 categories: mountain areas, isolated islands, and remote townships. In these areas, medical and care services are legally allowed to be practiced remotely according to the law and regulation of the state. There are 48 remote townships that account for 44% of the area in Taiwan, while the 0.36 million residents only account for 1.6% of the total population.

There is evidence that CH can increase access to services across a range of medical specialties without detrimental effects and improve opportunities for professional development [3]. However, most of these measures are temporary responses to government policy, and the sustainability of such services is an issue that must be addressed. Consequently, although technological interventions have improved accessibility, efficiency, and cost-effectiveness, the health status of rural residents remains a matter of concern [4]. Thus, the parameters of effective CH implementation in rural and remote contexts need to be explored to suggest strategies to manage issues appropriately.

Research suggests that the maturity of information communication technology, advances in health care, and the integration of health and social care may offer the fundamental infrastructure to boost the CH ecosystem [5]. Taiwan was selected as an ideal research area as it fulfills these requirements. Therefore, the results of this investigation may inspire those who are attempting to manage the issues of aging populations in a remote setting through a knowledge-sharing perspective.

To understand the obstacles faced in the implementation of CH in remote areas, this study explored the developmental barriers of CH in remote areas based on four representative cases in Taiwan. Geographically, CH in Taiwan has been divided into 4 parts: North, middle, South, and East. Each part has a designated hospital to support other hospitals and health centers in their areas. This study aimed to determine ideal strategies and methods by exploring the unknowns to discover insights for those facing similar issues in applying CH.

In this study, CH is defended as a technological solution that reshapes the direction of health care to be more proactive, preventive, and precisely targeted, and thus, more effective. CH provides great value in managing and preventing chronic diseases that result in tremendous burden on health care and social services. Stakeholder analysis was employed to facilitate strategy formation, as it can generate knowledge about how the characteristics of stakeholders influence decision-making processes as well as the relevant actors’ behavior, intentions, and interrelations.

### Theoretical Basis

**Figure 1:**
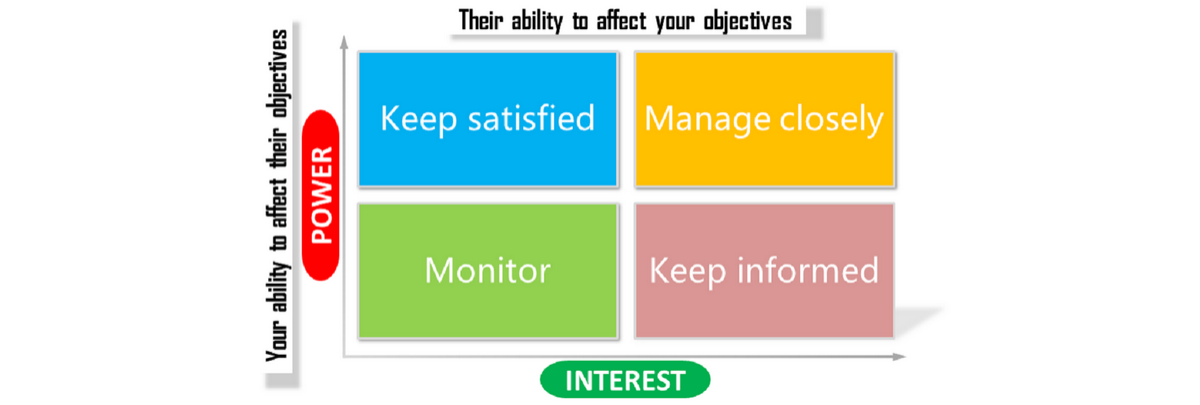
A simple stakeholder management model (adapted from Freeman and Reed [10]).

The challenges of accessing appropriate health services and of recruiting and retaining staff constrain the quality of health care in remote settings [6]. With respect to a proactive approach to current problems and insights into remote health, gaps remain [7]. Possible strategies for these issues include overcoming geographic isolation and facilitating rural and local health responses. This study explored how stakeholder characteristics influence the decision-making process. The literature suggests that the need to balance conflicts of interest and the influence of organizational factors and professional support may impact the quality of health in remote areas [8,9]. Therefore, this study explored perspectives on stakeholder arrangements, including the power and interests of stakeholders and their respective needs in the ecosystem, as shown in Figure 1.

Stakeholder analysis is often employed to facilitate the formation of strategies, as it can generate knowledge about how the characteristics of stakeholders influence decision-making processes as well as the relevant actors’ behaviors, intentions, and interrelations [8]. Courage, creativity, and a capacity to recognize opportunities for change will be key for public health advocates to create political incentives and manage political risks for leaders. Globalization may be helpful for leaders to gain awareness of the necessity of change and innovative strategies in health care [11]. Better insight into how health systems are structured and how they react over time to better adjust to health programs will also affect stakeholder management [12]. Despite considerable research on risk analysis in health politics, only little insight has been gained.

The value of stakeholder analysis in the health promotion domain is its ability to increase our understanding and capacity to participate in and contribute to health policy development by mapping the relevant actors. Once insights into their position and connection in networks are discovered, strategic planning can then be proposed [13].

General agreement among stakeholders may help the health information system advance in meeting the objectives of improving the governance, efficiency, user and provider satisfaction, and long-term fiscal sustainability of the health care system [14,15]. A stakeholder analysis framework is often used to highlight how key relationships with stakeholders may change with the perceived credibility of the organizational leaders and the legitimacy of their actions, as CH is a disruptive innovation in health care services. It is fascinating to explore how small rural health centers and a well-established, federally qualified community health center contribute to managing the risks of health care [16]. The concept of stakeholder management can help us to understand the tasks and structural changes via the legislation of decision makers [10]. Considerable systematic planning for health promotion that addresses people’s lives, work, and leisure can optimize health interventions for specific contextual contingencies and target crucial factors in the organizational context that influence behavior [17].

The rise of the patient-centered concept has increased the complexity of the health care system, which motivates partnerships between the public sector and private actors in stakeholder management [18]. The literature indicates that a situation with a multitude of actors with diverse interests suggests a loosely connected network, for those who attempt to influence policy will need to work with international-level, federal-level, and regional-level actors because they play an important role in bridging and connecting the decentralized regional-level and local-level actors as well as in initiating policy engagement and change [19,20]. Multicriteria decision analysis can add value to the strategic decision-making process in health technology assessment through systematic reviews, as it is more focused on how to engage stakeholders than to explain how to develop the algorithms and methodologies [21]. Analyzing routinely captured health information and giving feedback to clinical staff have been proposed to deliver better outcomes for patients and communities in the CH program [22].

The attempt to identify factors affecting the availability, accessibility, and coordination of services serves to develop and implement culturally sensitive service delivery in remote health care settings. Findings could inform recommendations for the provision of health services to contribute to the broader knowledge of rural and remote health service provision [23]. Access to allied health services is always an issue for people with disabilities living in rural and remote areas. Evidence indicates that CH is a valid option to provide those with disabilities alternative options to receive health care services [24]. Investigation of frail older adults and their stakeholders suggests that an integrated system with a care coordinator to improve connections between services and patients is urgently needed. It is a must to reduce bureaucracy and increase the timeliness of treatment and care. Measures to improve access to health and social care systems for pre-frail and frail patients, as well as their caregivers, must be considered [25]. Also, CH is beneficial for patients with chronic conditions as well as for the frail, by improving access to health care services and allowing them to be monitored at home [26].

Strengthening the rural deployment of stakeholders may attract and motivate graduates to work in rural areas, although the factors influencing recruitment of health professionals are varied [27]. If individual knowledge of successful aging and the associated economic outcomes, such as financial planning for retirement wellbeing, are to be harnessed, stakeholder management can help put pressure on the state to improve the current health insurance system, making it possible to offer a universal social pension that prioritizes people deprived of income due to a disability, severely debilitating disease, or lost work opportunity during their younger years [28].

### Research Questions

This study aimed to determine how geographical isolation and aging in place can be accommodated through CH stakeholder management based on the knowledge of the stakeholders’ identities and their interests and power in remote areas of Taiwan.

## Methods

**Figure 2:**
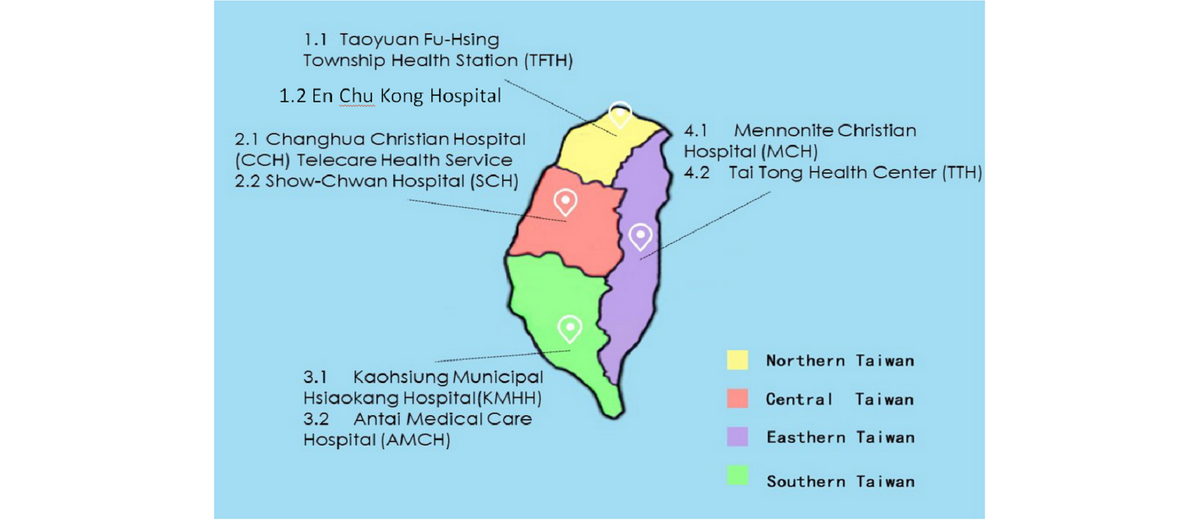
Remote health settings in Northern, Central, Southern, and Eastern Taiwan.

An exploratory approach was conducted involving semistructured interviews with 22 participants from 8 remote allied case studies [29]. The interviews explored perspectives on stakeholder arrangements, including the power and interests of stakeholders and the needs of all the parties in the ecosystem [30,31]. To manage health challenges for the aging population, the Taiwanese government divided Taiwan into 4 parts to conduct a pilot study of CH in 1996 (Figure 2). This provided the foundation for the current infrastructure of CH and smart health in Taiwan. Therefore, this study organized research according to this foundation. An exploratory approach was conducted involving semistructured, in-depth interviews with 22 participants in four remote allied case studies: Northern Taiwan, Central Taiwan, Southern Taiwan, and Eastern Taiwan (Figure 2). Northern Taiwan is represented by 1.1 Taoyuan Fu Hsing Township Health Station and 1.2 En Chu Kong Hospital; Central Taiwan by 2.1 Changhua Christian Hospital Telecare Health Service and 2.2 Show-Chwan Hospital; Southern Taiwan by 3.1 Kaohsiung Municipal Hsiaokang Hospital and 3.2 Antai Medical Care Hospital; and Eastern Taiwan by 4.1 Mennonite Christian Hospital and 4.2 Tai Tong Health Center.

This study investigated how geographical isolation and aging in place can be accommodated through CH stakeholder management with reference to, but not limited by, the interview questions outlined in Textbox 1, based on a qualitative method with focus groups.

**Interview questions used with the focus groups.**

1. Who are your stakeholders?
2. How do they influence your business?
3. Do you have cooperative agreements with other organizations?
4. Are these agreements offer you competitive advantages?

### Ethics

Ethical approval was obtained from the Ethics Committees of Ulster University (reference no RG3 RMcAdam2). Participants gave verbal consent according to the ethical guidelines.

### Participant Selection

The authors selected 8 allied case studies from 4 geographical areas covering all remote areas in Taiwan based on a literature review and the foundation of the governmental pilot schemes referred to in Figure 2. Participants and institutions were chosen accordingly. The participants included those who were responsible for CH projects; patients were excluded due to ethical concerns. Residence in the four administrative areas based on geographic and rural location was the main criterion for selecting participants, and experience with CH was the second selection criterion. These sampling criteria were chosen in order to include the inputs of major and significant participants with various levels of rural experience and management. Consequently, 8 health institutions with 22 participants that represented all remote areas of Taiwan were selected, as shown in Figure 2 and Table 1.

**Table 1:** Allied health professionals participating in the study.

Geographic area and health care institutions			Role and number of participants		Age (years), range
**1. North**					
	1.1 Taoyuan Fu Hsing Township Health Station		1 GP^a^, 1 nurse, 1 civil servant		40-50 (GP and civil servant), 30-40 (nurse)
	1.2 En Chu Kong Hospital		1 nurse		30-40
**2. Central**					
	2.1 Changhua Christian Hospital (CCH) Telecare Health Service		4 nurses		30-40
	2.2 Show-Chwan Hospital		4 nurses		30-40
**3. South**					
	3.1 Kaohsiung Municipal Hsiaokang Hospital		1 GP, 1 pharmacist		30-40
	3.2 Antai Medical Care Hospital		1 nurse, 2 GPs		30-40 (nurse), 40-50 (GPs)
**4. East**					
	4.1 Mennonite Christian Hospital		1 nurse, 1 IT^b^ director		40-50
	4.2 Tai-Tong Health Centre		1 GP, 2 social workers		25-45

^a^GP: general physician.

^b^IT: information technology.

### Data Collection

Following informed consent, researchers conducted face-to-face interviews with 22 participants lasting from 30 minutes to 2 hours. To ensure the significance, validity, and rigor of this qualitative research, several research strategies were conducted regarding the quality of data collection. These protocols include the use of the agile method to allow interviewees’ opinions to be validated and exchanged through a semistructured interview manner. Any differences between the interviews were discussed until consensus was reached. Interviews were audiotaped and transcribed verbatim by an independent typist with subsequent validation by the researcher. Meaningful quotations were adduced to represent important themes. To ensure participant confidentiality, data were de-identified before processing.

### Data Analysis

In this study, data were categorized using stakeholder and thematic analyses. Stakeholder analysis is conducted according to the degree of the stakeholders’ ability to affect health care providers’ objectives and health care providers’ ability to affect stakeholders’ objectives. It follows 4 steps to investigate how health institutions influence stakeholders for their interests: (1) identify stakeholders, (2) assess their interests and influence, (3) develop a communication management plan, and (4) engage and influence stakeholders. In the thematic analysis, codes were derived from the data through several steps including data cleaning, data summarizing, data analysis, and data mining. The categorization of data was continually revisited and reviewed until the themes and categories used to summarize and describe the findings were verified and accurately reflected the data. A qualitative data management system, NVivo 12 (QSR International, Melbourne, Australia), was employed to manage the data throughout the process. First, data were cleaned through an integration process to merge different terms with the same meaning. Thus, CH might be called “remote health,” “telehealth,” or “telecare” in the interviews, and these terms were merged according to the actual meaning of the interviewees. Second, the data were summarized, clustered, and categorized based on the interviewees’ meaning. Third, the data were analyzed and extracted according to the interviewees’ meaning at the stage of data mining. For example, some issues were raised by interviewees, but the meanings and root causes of the issues needed to be analyzed.

## Results

### Participants

A total of 22 participants representing 8 CH groups that covered all the remote areas in Taiwan were involved in this study. The majority of the participants were health professionals such as nurses (12/22, 54%), pharmacists (1/22, 5%), and general physicians (5/22, 22%), accounting for more than three-quarters of the sample. In addition, social workers accounted for approximately 10% (2/22, 9%) of the sample. Information technology professionals and administrators each accounted for 5% of the participants (1/22, 5% each), as shown in Multimedia Appendix 1.

### Stakeholder Analysis

Stakeholder analysis was conducted according to the degree of the health care providers’ ability to affect their stakeholders’ objectives and interests. Four steps of stakeholder management were used: (1) identify stakeholders, (2) assess their interests and influence, (3) develop a communication management plan, and (4) engage and influence stakeholders. Due to the complexity of the data, these steps are described in Multimedia Appendices 1-4. The results revealed the identity of the stakeholders of CH in remote areas, how they influence practices, and how associated agreements bring competitive advantages.

The stakeholders for 8 participating health facilitators were identified (Multimedia Appendix 1). These stakeholders included industrial players (software developers, hardware manufacturers, total solution providers, network providers), users and their associates (end users), government sectors, and academic researchers, which collectively constitute an ecosystem of CH. The industrial players included both software developers and total solution providers (eg, Fora Care Inc, Asus Cloud, Huede Technology, and Far EasTone Telecommunications), while some telecommunications companies fell in both categories of network providers and software developers (eg, Far EasTone Telecommunications and Chunghwa Telecom). This fact may suggest that the CH industry is a multidisciplinary domain. What is noticeable is that not all the health facilitators had a telehealth or telecare center; therefore, some had to rely on industrial players to analyze and manage collected data. Compared with public health facilitators, private health facilitators have different attitudes toward government sectors. Although all the end users were from remote areas, they could originate from mountain areas, remote townships, or isolated islands, which differentiates the nature of their health facilitators.

In Multimedia Appendix 2, the assessment of the interests of each CH stakeholder group and the influence of health facilitators is described. Government sectors are concerned with how to identify significant outcomes to increase and promote political publicity. For academic researchers, innovative topics for research and how to explore unknown and novel concepts to contribute to the body of knowledge seem to be of the utmost importance. End users are looking for user-friendly, efficient, and cost-effective solutions to manage their health care according to health facilitators. However, they may care more about their financial health than their physical health. Although most industrial players are interested in increasing sales through developing and perfecting their products and services, the concepts of software developers and hardware manufacturers will vary due to the nature of their businesses. What is noticeable is that the influence on stakeholders determines the power and strategies of health facilitators.

According to the assessment in Multimedia Appendix 2, potential strategies for health facilitators to develop a communication management plan for stakeholders are shown in Multimedia Appendix 3. First, the communication constraints of health facilitators should be determined, followed by identifying the information to be communicated. Finally, the methodology for communications is determined, to facilitate the communications. The challenges for health facilitators include finding the right people to deliver the communication. The participants indicated that information technology professionals usually do not comprehend their requests and even if they do eventually understand them, they may not be able to make relevant decisions. Moreover, it is difficult to include interdisciplinary people who can accommodate supply and demand from various areas in the CH ecosystem. These considerations constrain communication between health facilitators and their stakeholders. Although the intent to fulfill the stakeholders’ interests by exchanging resources is helpful, the degree of the bargaining power of stakeholders differs to that of health facilitators. Regarding the information to be communicated, many health facilitators admit that they still struggle. It should be stressed that strategies for increasing the visibility of institutions can facilitate communication with stakeholders.

From the data presented in Multimedia Appendix 3, the benefits of increasing the profile of health facilitators are clear. Regarding the next phase, Multimedia Appendix 4 provides information about how health facilitators can engage and influence their stakeholders according to the developed communication management plan. Then, the actual plans and strategies put in use are included. Health facilitators might influence stakeholders according to their interests. The participants indicated that industrial players offer free CH samples to receive test feedback from remote areas, especially mountain areas, because the features of geographic isolation and the local authority of general physicians (GPs) could significantly contribute to the development of a considerable number of products that could be brought to the market. After health facilitators achieve a solid reputation in the CH ecosystem, many opportunities for cooperation will spontaneously appear. Although the participants suggested that end users are usually concerned about the affordability of CH products and services, GPs may play an important role in educating patients about the value of CH. Other health facilitators, such as nurses, usually offer alternative solutions for patients to consider. Overall, those who have a typical environment or samples for CH to implement have stronger bargaining power when dealing with industrial players.

**Figure 3:**
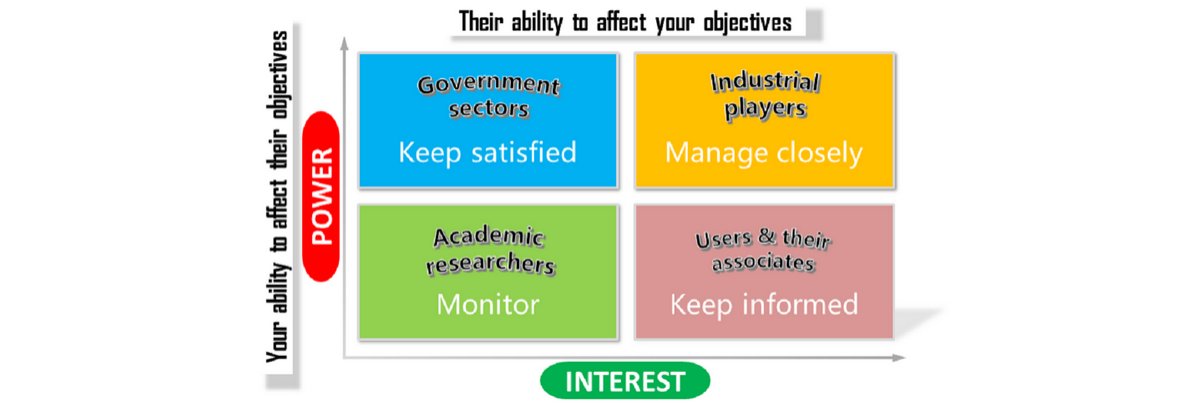
Stakeholder analysis of remote health care (source: adapted from Freeman and Reed [10]).

As mentioned, stakeholders representing government sectors, industrial players, academic researchers, and end users and their associates. The stakeholder analysis is mapped in Figure 3.

Initially, industrial players have relatively high importance, as they possess both technologies and economic benefits. Health providers in remote areas should, therefore, manage these closely. Next, government sectors are essential as they are key for funding the remote services as well as implementing rules and regulations. Remote users and their associates are less powerful in the remote health ecosystem than the parties in the first two categories, as they are usually voluntary and have limited economic strength. They are to be kept informed of any updates. Finally, academic researchers are less influential in affecting health care providers’ objectives. Thus, they play the role of serving as a reference.

Governmental funding is important as we have limited capability to commercialize remote health care in the initial stage…

The main purpose of offering remote health care is to address the social equality of health care rather than earning money.

Although remote areas lack resources, the geographic features can be ideal locations for industrial players to develop and test their products and services.

It is essential to keep connected to the latest health information and progress as well as global trends through academic interactions.

#### Full Disclosure of Conflicting Interests

Several themes, such as government interests and power of decision making were corroborated across remote services. These have been broadly grouped into the disclosure of conflicts of interest, asymmetry in decision making, and data development for risk assessment. Health care stakeholders are criticized for finding it hard to reach common ground, as they all have different interests. Industrial players tend to emphasize economic benefits rather than ethics and user privacy, while health care providers value security, ethics, and patient rights above all. Government sectors are concerned with political forces, and academic researchers care most about the social impact.

Government is propaganda…they only care about their political achievements.

We care about remote residents’ lives, feelings, and rights to receive care.

The population of remote areas is low; therefore, many businesses tend to ignore residents’ interests as their scale is too small to yield economic benefits.

A successful experience building up remote health models in Taiwan and expanding them worldwide may contribute significant impacts globally.

Although stakeholders in the remote health care ecosystem have various interests, they need to rely on each other to survive. Health providers need government’s support in establishing infrastructure and initial funding; industry’s technologies and services are needed in operating remote offerings alongside academia’s publicity and management’s inputs to increase their reputation and profile-raising. However, health providers in remote areas usually have little negotiating power in pricing and budget. Therefore, the skill of translating risks into opportunities is desirable to obtain benefits.

Big companies offer quality products to our telehealth center for the sake of our hospital; otherwise, they don’t care much about small businesses.

In remote areas, the infrastructure is poor, as the main telecom companies cannot gain profit from building it. It creates a challenge to practicing remote health even though it is essential for residents.

Most of our infrastructure relies on government funding.

Government will be happy to support us as long as the practice can show benefits to their political achievements.

Many main industrial players have difficulty meeting customers’ needs and designing user-friendly products; therefore, they come to us with free products to obtain testing samples in remote settings.

#### Asymmetry (Irregularity) in Decision Making

Although remote health care focuses on serving rural residents, their capability of decision making mainly depends on health care professionals, industrial players, and government.

Physicians have authority over patients, so their suggestions can strongly influence patients’ decisions.

If no remote infrastructure and services from industrial players, no connected health can be set up, but unfortunately, remote residents have no word in decision making.

As rural residents often are older, of low socioeconomic status, and poor health status and have limited purchasing power, their interests are safeguarded by government and health professionals. Fortunately, as they live in a democratic society, they can wield influence on the government through voting, which provides incentive for the government to invest in remote infrastructure – to earn political capital.

Although remote residents have little power and influence on the CH setting, their voice is often heard through GPs and other health facilitators: *“*Remote residents pay the same health insurance fees to the government; however, they hardly enjoy any health benefits due to poor infrastructure. It is unfair to them.”

It takes effort for GPs in remote areas to fight for residents’ rights. In the beginning, outcomes are limited, but little by little, their efforts have produced some fruit.

We have tried to contact the main telecom company; however, they show no interest in building infrastructure here as there are no economic benefits.

Luckily, we obtained support from the Minister, so that we can have some progress in remote services.

Thanks to the attention of the Minister, we got attention through publicity, and industrial people contacted us to discuss cooperation opportunities by offering free products for testing.

#### Data Development for Risk Management

To advance the remote health decision-making process, there is a need to think strategically about how data can inform risk, as the challenges of delivering CH in remote areas can travel in two directions. The upside of this challenge may generate better outcomes, but conversely, the downsides of the outcomes can be worse than expected. Geographic isolation and poor health resources may be downsides for CH, yet strategic planning about stakeholder management may maximize opportunities for gain, which may provide entrepreneurial opportunities as companies make money by converting challenges to opportunities. What distinguishes challenges and opportunities is therefore central to business success. Some challenges are having an adverse or positive effect on an organization’s profits from overseas activities. Talent that can integrate and facilitate resources to optimize the interests of CH’s stakeholders is required.

Practicing CH in remote areas does offer many opportunities. There are many examples, including boosting the development of high technologies, offering indications for health care policy making, and shortening the gaps in health implementation.

Our experience with CH in remote areas has not only contributed to many publications but also raised the profile of our hospital.

The successful experiences of practicing CH may be the best advertisement and marketing strategy for our hospital.

Taiwan’s geographic features are significant in practicing CH, as it is a representative case study…if CH devices can serve in such a challenging setting as Taiwan’s remote areas, there will be no issues in using it all over the world.

To move forward, a new decision paradigm is needed with the flexibility to consider new insights and scientific information. This approach would not create an environment in which the discussion of risk based on the information is avoided. Currently, although most state and federal regulations are not designed to protect individuals, they protect the public without defining what the public is or how many individuals constitute the public. As part of a new paradigm, researchers and policymakers should carefully consider whether current federal regulations are in fact designed to adequately protect individuals, especially those in vulnerable subpopulations. Any procedural change is an opportunity to engage stakeholders on how these regulations are structured to address these populations and in what contexts. Finally, the paradigm should incorporate evaluation in the decision-making process, as assessing the impact of a decision is vital to the success of future decision making.

## Discussion

### Principal Findings

This study aimed to identify how geographical isolation and aging in place can be accommodated through CH stakeholder management based on identification of the stakeholders and their interests. We determined the interests of stakeholders and strategies that health facilitators can employ. Industrial players look for participants to test their products and a CH environment to comprehensively develop products for better sales. Governments seek political achievement, and academic researchers pursue interesting topics and data to have an impact society. Offering benefits that can fulfill stakeholders’ interests will help to overcome the challenge of resource shortages in remote areas. However, it is not a “one-size-fits-all” solution. Health facilitators should recognize their competitive advantages to differentiate their strengths from those of others. Once their uniqueness can meet their supplier’s need, they are able to exchange benefits to address their shortages.

Compared with current and past literature, our findings offer further insights into stakeholder engagement. The knowledge foundation of this study was developed from a current understanding of stakeholders’ perspectives from the literature. The importance of the identification of stakeholders in electronic health has been reported [32,33]. Based on this, the concept of the co-creation and co-design of a health facility with stakeholders has been discussed [34,35]. Perspectives on a patient-centric model include an increase in technology adoption [36]. Strategies for successful implementation of technology for aging in place have been studied [37]. Person-centeredness, clinician acceptability, and informatics feasibility have been achieved and ensured through technology applications [35].

Regarding the research context, most previous literature has focused on the health care system in Western or developed countries where remote areas are numerous and internet infrastructures are better developed [4,38-41]. These countries encounter the challenges of an aging population earlier than developing or underdeveloped countries. In contrast, stakeholder perspectives have not been extensively studied in some regions in Asia that are experiencing a similarly aging population and are interested in developing CH. Taiwan can be seen as a typical case; its rate of aging and degree of advanced technology are as high as those in Western countries [3,42-44]. Additionally, Taiwan has representative remote areas and developed health care performance [3,43]. Therefore, stakeholder engagement in CH should be of interest to improve the challenges with resource shortages in remote areas. Moreover, Taiwan has an integrated health and care system, which can facilitate health information sharing and social interaction more effectively [45].

### Possible Bias in Participant Selection

Bias should be prevented to ensure the validity and value of research. However, it is difficult to avoid bias due to its complex factors. Bias can occur from the research environment, participants, and even the researchers themselves. Some researchers may intentionally influence participants to obtain the results expected. Moreover, some bias from researchers occurs unconsciously, which makes it more difficult to prevent. This phenomenon is especially present in qualitative research because qualitative research relies more on the experience and judgment of the researcher. Also, the type of data collected is subjective and unique to the person or situation. Therefore, it is much harder to avoid bias in qualitative research than in quantitative research.

Recognizing that bias exists in all research may be a good start to avoid bias. Then, the researcher should be informed about potential bias so he or she can avoid bias as much as possible. Before the research has been conducted, design bias can occur. Omission bias from selection or sampling may also occur during the research. If the study is not well planned, inclusive bias can occur when researchers look for quick solutions and convenient options.

To avoid bias, the researcher first checked the guidance for qualitative research. Second, the interview plan was discussed with the supervisor at various times to maintain the objectiveness of the interview questions. Indirect and open-ended questions were asked to allow information to flow more freely. Third, all the interviews were recorded and analyzed without personal preference. Finally, independent reviewers were invited to review the research to help maintain objectivity in the research.

### Validity

In terms of the validity of the data analysis, this study followed rigorous qualitative research principles to ensure the credibility, transferability, and reliability of the data [29]. The triangulation approach was employed as a testing method to examine the validity of research [30,31]. It helps to capture different dimensions of the same phenomenon. In this study, research data triangulation and investigator triangulation were adopted to test the validity and reliability of research. In the research data triangulation, the interview questionnaires were discussed using the Delphi method with 8 focus groups and analyzed using NVivo software. In the investigator triangulation, multiple investigators, such as health professionals and experts, were invited to evaluate and review the data for selective perception and illumination blind spots in an interpretive manner to understand multiple ways of seeing the data.

### Strengths

This study addressed a topic that had previously not been well studied so that it may offer insight for future projects on CH in rural areas. Different from other case studies in which the research focused mainly on developed countries, this study considered a region between developed countries and developing countries in order to discover methods for accommodating the resource shortage in remote areas. This study considered Taiwanese CH as a case study, as it meets all the CH preconditions and essential requirements. Not only are advanced technology and medicine present but the ecosystem for boosting CH is also complete and comprehensive in Taiwan. Recently, Taiwan integrated social care with its health department, to become a health and social welfare department. Chronic conditions are prevalent due to an aging demographic. Geographically, Taiwan has populations in urban areas and in many remote areas and isolated islands. Moreover, it is a mixture of public and private health care systems. These features suggest that if CH practice can be successfully applied in Taiwan, it is likely also to suit other countries around the world.

### Limitations

A limitation of this study is that only one case study approach was used, making it difficult to generalize the findings due to a limited sample size. Generic principles and popular interpretations are not easily formed because the purpose of the study was to analyze problems rather than obtain summarized or statistical data. Consequently, certain inductions might be arbitrary and subjective. In addition, technical limitations and researcher bias may be an issue because a standard data analysis method was not used; therefore, the data interpretation and presentation of evidence are influenced by the researcher’s choice, which may affect the results of the study. Further generalization is likely to require additional case studies for different potential contexts, which could include diverse case studies that may show cultural differences and influences.

### Future Research

The outcomes of this qualitative research can serve as preliminary quantitative research on the degree of the influence of health facilitators on stakeholders’ interests. Alternatively, the optimization of two or multiple objectives using a quantitative method can be applied to discover the optimal interests of all stakeholders in future work.

### Conclusions

This study contributes to current knowledge by exploring the features of CH in remote areas and investigating its implementation from the perspectives of stakeholder management. Methods to accommodate geographic isolation and aging in place through CH stakeholder management are discussed based on the identification of the stakeholders and their interests and power in remote areas of Taiwan. The results offer insights for managing remote health through a CH platform, which can be used as preliminary quantitative research. Consequently, these findings could help more effectively facilitate diverse stakeholder engagement for health information sharing and social interaction.

## Appendix

Multimedia Appendix 1 Connected health stakeholder identification.

Multimedia Appendix 2 Connected health stakeholders’ interests and influence assessment.

Multimedia Appendix 3 Connected health stakeholders’ communication management development.

Multimedia Appendix 4 Stakeholders’ engagement and influence.

Multimedia Appendix 5 Interview questions template.

## Abbreviations

CH: connected health
GP: general physician
IT: information technology
