# Supplementary material for: How Geographical Isolation and Aging in Place Can Be Accommodated Through Connected Health Stakeholder Management: Qualitative Study With Focus Groups
Source: J Med Internet Res. 2020 May 27;22(5):e15976. doi: 10.2196/15976 (PMC7287745; doi:10.2196/15976)
Supplement: Multimedia Appendix 1 [file jmir_v22i5e15976_app1.docx]

Appendix 1. CH’s stakeholder identification

|  |  | Stakeholders | | | | | | | |
| --- | --- | --- | --- | --- | --- | --- | --- | --- | --- |
|  |  | Industrial players | | | | | Users & their associate | Government | Academia |
| 1. Identify stakeholders | CH facilitators in remote areas | 1. Software developers | 2. Hardware manufacturers | 3. Total solutions providers | 4. Connected health care service providers | 5. Network providers | 6. End users | 7. Government sectors | 8. Academia |
|  | 1.1 Taoyuan Fu Hsing Township Health Station, | Hospital Information System (HIS)/Picture Archiving and Communication System (PACS) | Qisda Corporation | (HIS)/(PACS) | Taipei Medical University TH and Telecare Center | Chunghwa Telecom | Residents in mountain areas and remote townships | Health and Welfare Department | Government Institutions / Universities/Industrial Technology Research Institute (ITRI)/Insurance companies |
|  |  |  |  |  | Chang Gung Medical Foundation |  |  | National Center for Higher-performance Computing for Cybersecurity |  |
|  | 1.2 En Chu Kong Hospital | Fora Care Inc. | TaiDoc /Fora Care | Fora Care Inc. | En Chu Kong Hospital Community Medicine | Chunghwa Telecom | Residents in remote townships | Health and Welfare Department |  |
|  | 2.1 Changhua Christian Hospital (CCH) Telecare Health Service | Huede Technology | Huede Technology | Huede Technology | Changhua Christian Hospital (CCH) Telecare Health Service | Chunghwa Telecom | Residents in remote townships | Health and Welfare Department |  |
|  |  |  | Apexbio |  |  |  | Residents in mountain areas |  |  |
|  |  | Fora Care Inc. | Far EasTone | Far EasTone Telecommunications |  |  |  |  |  |
|  | 2.2 Show-Chwan Hospital, Southern Taiwan | Asus Cloud | TaiDoc/Fora Care | Asus Cloud | Show-Chwan Hospital Telehealthcare center | Chunghwa Telecom | Residents in remote townships | Health and Welfare Department |  |
|  |  |  | OSTAR |  |  |  |  |  |  |
|  | 3.1 Kaohsiung Municipal Hsiaokang Hospital | ASUS HealthCare | Apexbio | Far EasTone Telecommunications | Kaohsiung Municipal Hospital | Far EasTone Telecommunications | Residents in mountain areas | Health and Welfare Department |  |
|  | 3.2 Antai Medical Care Hospital | (HIS)/(PACS) | GE | (HIS)/(PACS) | N/A | Chunghwa Telecom | Residents in remote townships | Health and Welfare Department |  |
|  | 4.1 Mennonite Christian Hospital Telecare center | CHT/Far EasTone Telecommunications | Roche | Far EasTone Telecommunications | Mennonite Christian Hospital Telecare center | Chunghwa Telecom | Residents in mountain areas, isolated islands, and Residents in remote townships. | Health and Welfare Department |  |
|  |  |  | Apexbio |  |  |  |  |  |  |
|  | 4.2 Tai Tong Health Centre | Far EasTone Telecommunications | Apexbio | Far EasTone Telecommunications | Far EasTone Health | Chunghwa Telecom/ Far EasTone Telecommunications | Residents in mountain areas, isolated islands, and Residents in remote townships. | Health and Welfare Department |  |
|  |  |  |  |  |  |  |  | National Center for Higher-performance Computing for Cybersecurity |  |
