# Supplementary material for: How Geographical Isolation and Aging in Place Can Be Accommodated Through Connected Health Stakeholder Management: Qualitative Study With Focus Groups
Source: J Med Internet Res. 2020 May 27;22(5):e15976. doi: 10.2196/15976 (PMC7287745; doi:10.2196/15976)
Supplement: Multimedia Appendix 2 [file jmir_v22i5e15976_app2.docx]

Appendix 2. CH stakeholders’ interests and influence assessment

| 2. Assess their interests and influence |  |  | Stakeholders' interests | | | | | | | |
| --- | --- | --- | --- | --- | --- | --- | --- | --- | --- | --- |
|  |  |  | Industrial players | | | | | Users & their associate | Government | Academia |
|  |  |  | 1. Software developers | 2. Hardware manufacturers | 3. Total solutions providers | 4. Connected health care service providers | 5. Network providers | 6. End users | 7. Government sectors | 8. Academia |
|  |  |  | Develop better software solutions to increase products' competitiveness. | Look for suitable hardware applications in healthcare sectors to increase sales. | Look for suitable opportunities to integrate software and hardware to increase sales. | Look for better solutions and interface to increase in efficiency and cost-effectiveness. | Look for cost-effective businesses and sales. | Look for user-friendly, efficient and cost-effective solutions to manage their healthcare. | Look for good outcomes to increase and promote politic publicity. | Look for innovative topics to research and explore unknown and novelty to contribute the body of knowledge. |
|  | Participants' power | 1.1 Taoyuan Fu- Hsing Township Health Station, | Offer significant experimental environment to test products and services. | | | | | Potential to influence users' decision making. | Potential to become an example to demonstrate government 's achievement. | Potential to offer valuable data for research. |
|  |  | 1.2 En Chu Kong Hospital | Offer some experimental cases to test products and services but limited cases due to low population. | | | | | Limited influence to end-users due to the nature of care. | Limited influence due to the nature of private sector. | Potential to offer valuable data for research but limited to sample numbers. |
|  |  | 2.1 Changhua Christian Hospital (CCH) Telecare Health Service | Offer significant experimental cases to test products and services. | | | | | Limited influence to end-users due to the nature of care. | Limited influence due to the nature of private sector. | Potential to offer valuable data for research. |
|  |  | 2.2 Show-Chwan Hospital | Offer significant experimental environment and cases to test products and services. | | | | | Limited influence to end-users due to the nature of care. | Limited influence due to the nature of private sector. | Potential to offer valuable data for research. |
|  |  | 3.1 Kaohsiung Municipal Hsiaokang Hospital | Offer low experimental cases to test products and services due to the numbers of cases. | | | | | Limited influence to end-users due to the nature of care. | Limited influence due to the nature of private sector. | Potential to offer valuable data for research but limited to sample numbers. |
|  |  | 3.2 Antai Medical Care Hospital | Offer low experimental cases to test products and services due to the numbers of cases. | | | | | Limited influence to end-users due to the nature of care. | Limited influence due to low significant in demonstrating political achievement. | Potential to offer valuable data for research but limited to sample numbers. |
|  |  | 4.1 Mennonite Christian Hospital Telecare center | Offer significant experimental cases to test products and services but limited cases due to low population. | | | | | Limited influence to end-users due to the nature of care. | Limited influence due to the nature of private sector. | Potential to offer valuable data for research. |
|  |  | 4.2 Tai Tong Health Centre | Offer significant experimental environment to test products and services. | | | | | Potential to influence users' decision making but still limited to the purchase capability. | Potential to become an example to demonstrate government 's achievement. | Potential to offer valuable data for research. |
