# Supplementary material for: How Geographical Isolation and Aging in Place Can Be Accommodated Through Connected Health Stakeholder Management: Qualitative Study With Focus Groups
Source: J Med Internet Res. 2020 May 27;22(5):e15976. doi: 10.2196/15976 (PMC7287745; doi:10.2196/15976)
Supplement: Multimedia Appendix 3 [file jmir_v22i5e15976_app3.docx]

Appendix 3. CH stakeholders’ communication management development

| Stakeholders management step 3 |  |  | Stakeholders' interests | | | | | | | |
| --- | --- | --- | --- | --- | --- | --- | --- | --- | --- | --- |
|  |  |  | Industrial players | | | | | Users & their associate | Government | Academia |
|  |  |  | 1. Software developers | 2. Hardware manufacturers | 3. Total solutions providers | 4. Connected health care service providers | 5. Network providers | 6. End users | 7. Government sectors | 8. Academia |
| 3. Develop communication management | Communication Management Focus | Healthcare Institutions | Develop better software solutions to increase compactivity. | Look for suitable hardware applications in healthcare sectors to increase sales. | Look for suitable opportunities to integrate software and hardware to increase sales. | Look for better solutions and interface to increase in efficiency and cost-effectiveness. | Look for cost-effective businesses and sales. | Look for user-friendly, efficient and cost-effective solutions to manage their healthcare. | Look for good outcomes to increase and promote politic publicity. | Look for innovative topics to research and explore unknown and novelty to contribute the body of knowledge. |
|  | 1. Determine communication constraints | 1.1 Taoyuan Fu Hsing Township Health Station, | 1. It's challenging to find the right people to communicate. 2. It is hard for people in different fields to understand each other's needs and focus. 3. Increasing the visibility of institutions to draw people's attention may be helpful for communications. | | | | | 1. Users' purchase capability tends to below. 2. Communicate the value of CH. 3. Use professional authority to influence decision making of users if requested. | 1. It's challenging to communicate with the government sector as the supervised institution without significant achievements. 2. Any potential achievement may be useful information to be communicated. 3. Take government's annual visiting or reporting opportunities to communicate with them. | 1. There is little interaction between Health institution and Academia unless there is a need for conducting research. 2. Use research topic as communication context. 3. Use supply and demand method from each side in the research to communicate. |
|  | 2. Determine Information to be communicated | 1.2 En Chu Kong Hospital | 1. The bargaining power to the supplier is low due to limited samples. 2. It is hard for people in different fields to understand each other's needs and focus. 3. Increasing the visibility of institutions to draw people's attention may be helpful for communications. | | | | | 1. Users' purchase capability tends to be low. 2. Communicate the value of CH. 3. Offer CH services as alternative solutions for users to choose. | 1. It's challenging to communicate with the government sector as a private institution. 2. Any potential achievement may be useful information to be communicated. 3. Take government's annual visiting or reporting opportunities to communicate with them. | 1. There is little interaction between Health institution and Academia unless there is a need for conducting research. 2. Use research topic as communication context. 3. Use supply and demand method from each side in the research to communicate. |
|  | 3. Determine Methodology for communications | 2.1 Changhua Christian Hospital (CCH) Telecare Health Service | 1. It's challenging to persuade the value of CH services and to overcome the issue of low purchase capability of remote residents. 2. It is hard for people in different fields to understand each other's needs and focus. 3. Increasing the visibility of institutions to draw people's attention may be helpful for communications. | | | | | 1. Users' purchase capability tends to be low. 2. Communicate the value of CH. 3. Offer CH services as alternative solutions for users to choose. | 1. It's challenging to communicate with the government sector as a private institution. 2. Any potential achievement may be useful information to be communicated. 3. Take government's annual visiting or reporting opportunities to communicate with them. | 1. There is little interaction between Health institution and Academia unless there is a need for conducting research. 2. Use research topic as communication context. 3. Use supply and demand method from each side in the research to communicate. |
|  |  | 2.2 Show-Chwan Hospital | 1. It's challenging to find the right people to communicate. 2. It is hard for people in different fields to understand each other's needs and focus. 3. Increasing the visibility of institutions to draw people's attention may be helpful for communications. | | | | | 1. Users' purchase capability tends to be low. 2. Communicate the value of CH. 3. Offer CH services as alternative solutions for users to choose. | 1. It's challenging to communicate with the government sector as a private institution. 2. Any potential achievement may be useful information to be communicated. 3. Take government's annual visiting or reporting opportunities to communicate with them. | 1. There is little interaction between Health institution and Academia unless there is a need for conducting research. 2. Use research topic as communication context. 3. Use supply and demand method from each side in the research to communicate. |
|  |  | 3.1 Kaohsiung Municipal Hsiaokang Hospital | 1. It's challenging to find the right people to communicate. 2. It is hard for people in different fields to understand each other's needs and focus. 3. Increasing the visibility of institutions to draw people's attention may be helpful for communications. | | | | | Limited influence to end-users due to the nature of care. | 1. It's challenging to communicate with the government sector as a private institution. 2. Any potential achievement may be useful information to be communicated. 3. Take government's annual visiting or reporting opportunities to communicate with them. | 1. There is little interaction between Health institution and Academia unless there is a need for conducting research. 2. Use research topic as communication context. 3. Use supply and demand method from each side in the research to communicate. |
|  |  | 3.2 Antai Medical Care Hospital | 1. The bargaining power to the supplier is low due to limited samples and significant contributions. 2. It is hard for people in different fields to understand each other's needs and focus. 3. Increasing the visibility of institutions to draw people's attention may be helpful for communications. | | | | | Limited influence to end-users due to the nature of care. | 1. It's challenging to communicate with the government sector as a private institution. 2. Any potential achievement may be useful information to be communicated. 3. Take government's annual visiting or reporting opportunities to communicate with them. | 1. There is little interaction between Health institution and Academia unless there is a need for conducting research. 2. Use research topic as communication context. 3. Use supply and demand method from each side in the research to communicate. |
|  |  | 4.1 Mennonite Christian Hospital Telecare center | 1. It's challenging to find the right people to communicate. 2. It is hard for people in different fields to understand each other's needs and focus. 3. Increasing the visibility of institutions to draw people's attention may be helpful for communications. | | | | | 1. Users' purchase capability tends to be low. 2. Communicate the value of CH. 3. Offer CH services as alternative solutions for users to choose. | 1. It's challenging to communicate with the government sector as a private institution. 2. Any potential achievement may be useful information to be communicated. 3. Take government's annual visiting or reporting opportunities to communicate with them. | 1. There is little interaction between Health institution and Academia unless there is a need for conducting research. 2. Use research topic as communication context. 3. Use supply and demand method from each side in the research to communicate. |
|  |  | 4.2 Tai Tong Health Centre | 1. It's challenging to find the right people to communicate. 2. It is hard for people in different fields to understand each other's needs and focus. 3. Increasing the visibility of institutions to draw people's attention may be helpful for communications. | | | | | 1. Users' purchase capability tends to be low. 2. Communicate the value of CH. 3. Use professional authority to influence decision making of users if requested. | 1. It's challenging to communicate with the government sector as the supervised institution without significant achievements. 2. Any potential achievement may be useful information to be communicated. 3. Take government's annual visiting or reporting opportunities to communicate with them. | 1. There is little interaction between Health institution and Academia unless there is a need for conducting research. 2. Use research topic as communication context. 3. Use supply and demand method from each side in the research to communicate. |
