# Supplementary material for: How Geographical Isolation and Aging in Place Can Be Accommodated Through Connected Health Stakeholder Management: Qualitative Study With Focus Groups
Source: J Med Internet Res. 2020 May 27;22(5):e15976. doi: 10.2196/15976 (PMC7287745; doi:10.2196/15976)
Supplement: Multimedia Appendix 4 [file jmir_v22i5e15976_app4.docx]

Appendix 4. Stakeholders’ Engagement and influence

| Stakeholders management step 4 |  |  | Stakeholders' interests | | | | | | | |
| --- | --- | --- | --- | --- | --- | --- | --- | --- | --- | --- |
|  |  |  | Industrial players | | | | | Users & their associate | Government | Academia |
|  |  |  | 1. Software developers | 2. Hardware manufacturers | 3. Total solutions providers | 4. Connected health care service providers | 5. Network providers | 6. End users | 7. Government sectors | 8. Academia |
| 4. Engage and influence stakeholders | Engage and influence stakeholders main points | Healthcare Institutions | Develop better software solutions to increase compactivity. | Look for suitable hardware applications in healthcare sectors to increase sales. | Look for suitable opportunities to integrate software and hardware to increase sales. | Look for better solutions and interface to increase in efficiency and cost-effectiveness. | Look for cost-effective businesses and sales. | Look for user-friendly, efficient and cost-effective solutions to manage their healthcare. | Look for good outcomes to increase and promote politic publicity. | Look for innovative topics to research and explore unknown and novelty to contribute the body of knowledge. |
|  | 1. How you need to influence each stakeholders? | 1.1 Taoyuan Fu Hsing Township Health Station | 1. Influence them according to their interests: offering a considerable research environment of CH for them to develop suitable products to the market. 2. This intention might be achieved through increasing its visibility through media and publications. 3. Promote the achievement in the media, magazine and on-line. | | | | | 1. General Physician can educate patients the concept of CH. 2. Offer alternative solutions for patients to use. 3. Negotiate free CH sample for patients to use to obtain feedback. | 1. Inform its supervisor the needs and potential impacts of implementing CH. 2. Offer opportunities and achievement for government to promote their political achievement.  3. Take government's annual visiting or reporting opportunities to communicate with them. | 1. Open to Academia for research. 2. Welcome interview opportunities. 3. Accept interviews from magazine and university researchers. |
|  | 2. How you might do this? | 1.2 En Chu Kong Hospital | 1. Influence them according to their interests: offering a considerable research environment of CH for them to develop suitable products to the market. 2. This intention might be achieved through increasing its visibility through media and publications. 3. Look for board of directors to support CH implementation and maintain good relationship with industrial players. | | | | | 1. Nurses can educate patients the concept of CH. 2. Offer alternative solutions for patients to use. 3. Persuade patients to use CH by revealing its value and competitive price. | 1. Inform government the needs and potential impacts of implementing CH. 2. Offer opportunities and achievement for government to promote their political achievement. 3.Fellow government's policy without proactive interaction. | 1. Open to Academia for research. 2. Welcome interview opportunities. 3. Accept interviews from magazine and university researchers. |
|  | 3. Make plans | 2.1 Changhua Christian Hospital (CCH) Telecare Health Service | 1. Influence them according to their interests: offering a considerable research cases of CH for them to develop suitable products to the market. 2. This intention might be achieved through increasing its visibility through media and publications. 3. Promote the achievement in the media, magazine and on-line. | | | | | 1. Nurses can educate patients the concept of CH. 2. Offer alternative solutions for patients to use. 3. Keep deliver care to patients and residents to build up reputations. | 1. Inform government the needs and potential impacts of implementing CH. 2. Offer opportunities and achievement for government to promote their political achievement. 3. Look for government projects and funding to implementing CH. | 1. Open to Academia for research. 2. Welcome interview opportunities. 3. Publish its outcomes through its researchers and visitors. |
|  |  | 2.2 Show-Chwan Hospital | 1. Influence them according to their interests: offering a considerable research cases of CH for them to develop suitable products to the market. 2. This intention might be achieved through increasing its visibility through media and publications. 3. Promote its achievement in the media, magazine and on-line to build up reputations in the industry. | | | | | 1. Nurses can educate patients the concept of CH. 2. Offer alternative solutions for patients to use. 3. Keep deliver care to patients and residents to build up reputations. | 1. Inform government the needs and potential impacts of implementing CH. 2. Offer opportunities and achievement for government to promote their political achievement. 3. Look for government projects and funding to implementing CH. | 1. Open to Academia for research. 2. Welcome interview opportunities. 3. Publish its outcomes through its researchers and visitors. |
|  |  | 3.1 Kaohsiung Municipal Hsiaokang Hospital | 1. Influence them according to their interests: offering a considerable research environment of CH for them to develop suitable products to the market. 2. This intention might be achieved through increasing its visibility through media and publications. 3. Promote the achievement in the media, magazine and on-line. | | | | | 1. General Physician can educate patients the concept of CH. 2. Offer alternative solutions for patients to use. 3. Offer patients its hospital networks to increase its competitiveness. | 1. Inform its supervisor the needs and potential impacts of implementing CH. 2. Offer opportunities and achievement for government to promote their political achievement . 3. Look for government projects and funding to implementing CH. | 1. Open to Academia for research. 2. Welcome interview opportunities. 3. Encourage outreach activities and conference attending. |
|  |  | 3.2 Antai Medical Care Hospital | 1. Influence them according to their interests: offering a considerable research environment of CH for them to develop suitable products to the market. 2. This intention might be achieved through increasing its visibility through media and publications. 3. Develop unique advantages in healthcare according to the features of hospital. | | | | | 1. General Physician can educate patients the concept of CH. 2. Offer alternative solutions for patients to use. 3. Deliver care to patients to build up its reputation. | 1. Inform its supervisor the needs and potential impacts of implementing CH. 2. Offer opportunities and achievement for government to promote their political achievement. 3. Look for government projects and funding to sustain hospital. | 1. Open to Academia for research. 2. Welcome interview opportunities. 3. N/A. |
|  |  | 4.1 Mennonite Christian Hospital Telecare center | 1. Influence them according to their interests: offering a considerable research environment of CH for them to develop suitable products to the market. 2. This intention might be achieved through increasing its visibility through media and publications. 3. Use the influence of its hospital to gain good deals for implementing CH. | | | | | 1. General Physician can educate patients the concept of CH. 2. Offer alternative solutions for patients to use. 3. Negotiate free CH sample for patients to use to obtain feedback. | 1. Inform its supervisor the needs and potential impacts of implementing CH. 2. Offer opportunities and achievement for government to promote their political achievement. 3. Look for government projects and funding to sustain hospital. | 1. Open to Academia for research. 2. Welcome interview opportunities. 3. Accept interviews from magazine and university researchers. |
|  |  | 4.2 Tai Tong Health Centre | 1. Influence them according to their interests: offering a considerable research environment of CH for them to develop suitable products to the market. 2. This intention might be achieved through increasing its visibility through media and publications. 3. Promote its uniqueness and in the media, magazine and on-line, such as Tedx, Youtube. | | | | | 1. General Physician can educate patients the concept of CH. 2. Offer alternative solutions for patients to use. 3. Raise its visibility through publication and media to fundraising for develop remote communities. | 1. Inform its supervisor the needs and potential impacts of implementing CH. 2. Offer opportunities and achievement for government to promote their political achievement. 3. Look for fundraising opportunities rather than getting government involved. | 1. Open to Academia for research. 2. Welcome interview opportunities. 3. Accept interviews from magazine and university researchers. |
