# Supplementary material for: How Geographical Isolation and Aging in Place Can Be Accommodated Through Connected Health Stakeholder Management: Qualitative Study With Focus Groups
Source: J Med Internet Res. 2020 May 27;22(5):e15976. doi: 10.2196/15976 (PMC7287745; doi:10.2196/15976)
Supplement: Multimedia Appendix 5 [file jmir_v22i5e15976_app5.docx]

**Appendix 5 Interview questions template**

1. What kind of role that technology plays in the healthcare sector from your point of view?

3. What about the technology adaptability of your customers?

4. What problems do you solve? And what opportunities do you exploit?

5. Who are your stakeholders and target customers?

Is the offer different for different segments?

How do you interact with stakeholders?

6. What resources can you use to run your business unique?

7. How can you benefit from Public policy in the developing of Connected Health/ remote health/ telehealth business?

8. What barriers do you encounter when you are promoting CH?

9. What are the barriers to healthcare innovation in remote areas?
